# Arsenic trioxide induces proteasome dependent TBLR1-RARα degradation to improve leukemia eradication through cell differentiation enhancement

**DOI:** 10.7150/jca.66175

**Published:** 2022-04-18

**Authors:** Yirui Chen, Manning Li, Han Wu, Shijin Yuan, Yan Xia, Yingjian Wang, Ye Peng, Jianping Lan, Yanzhong Wang

**Affiliations:** 1 Cancer center, Department of Hematology, Zhejiang Provincial People's Hospital, Affiliated People's Hospital, Hangzhou Medical College, 58 Shangtang Road, Hangzhou, Zhejiang, China, 310014.; 2 Department of Clinical Laboratory, Sir Run Run Shaw Hospital, Zhejiang University School of Medicine, 3 East Qingchun Road, Hangzhou, Zhejiang, China, 310016.; 3 Department of Clinical Laboratory, Xiasha Campus, Sir Run Run Shaw Hospital, Zhejiang University School of Medicine, 3 East Qingchun Road, Hangzhou, Zhejiang, China, 310016.

**Keywords:** Acute promyelocytic leukemia, TBLR1-RARα, Arsenic trioxide, Cell differentiation, Mitochondrial pathway apoptosis, Proteasome-mediated degradation

## Abstract

Background: Acute promyelocytic leukemia (APL) mainly harbors PML-RARα fusion gene, which is sensitive to all-trans retinoic acid (ATRA) and arsenic trioxide (ATO) treatment. However, APL harboring other RARα fusion genes exhibit different drug sensitivity. Here, we investigated the role and mechanism of TBLR1-RARα, a rare RARα fusion gene, on ATO treatment in leukemia cells.

Methods: By constructing two cell models of leukemia cell line HL-60 and U937 with overexpressed TBLR1-RARα, we detected the cell differentiation in the two cell models after ATO treatment by flow cytometry and Wright staining. Meanwhile, cell viability, colony formation and apoptosis were also determined after ATO treatment.

Results: We found that TBLR1-RARα enhanced ATO-induced apoptosis and cell proliferation inhibition. Besides, TBLR1-RARα also promoted ATO-induced cell differentiation. Furthermore, we found that the mitochondrial caspase pathway was involved in the apoptosis induced by ATO treatment in TBLR1-RARα positive leukemia cells. Moreover, ATO mediated TBLR1-RARα protein degradation via proteasome pathway, which accounts for the transcriptional activation of RARα target gene and is further involved in cell differentiation of TBLR1-RARα positive leukemia cells.

Conclusions: Our study provides evidence that TBLR1-RARα positive APL patients may benefit from ATO treatment, thereby improving the appropriate management in TBLR1-RARα positive APL.

## Introduction

Acute promyelocytic leukemia (APL) is a special entity of acute myeloid leukemia (AML), which accounts for approximately 10%-15% of AML 1. The majority of APL cases are characterized by t (15;17)(q24;q21) chromosomal translocation, resulting in the fusion between promyelocytic leukemia (PML) gene and the retinoic acid receptor alpha (RARα) gene 2. Harboring PML-RARα fusion gene, APL is sensitive to all-trans retinoic acid (ATRA) and arsenic trioxide (ATO) treatment, which turns APL from the most fatal subtype of AML into the most curable subtype of AML in the past decades 3. The treatment of APL is a typical paradigm of oncoprotein-targeted therapy. ATRA could lead roughly 80%-90% of newly diagnosed and relapsed APL patients achieving complete remission (CR) as a single agent. So, in the past decades, the combination of ATRA with chemotherapy has been used as first-line therapy. However, most of the patients could not achieve long-term remission after the combination of ATRA with chemotherapy 4. For the relapsed patients, ATRA plus ATO treatment was recommended for salvage therapy, which induced long-term remission and cured around 50-81% of relapsed APL patients 5. Recently, ATO has been approved for the new first-line treatment of newly diagnosed patients with low-to-intermediate risk APL 6. Clinical trial in *de novo* APL patients showed that the combination of ATO with ATRA led to a CR rate of 90-100% in newly diagnosed and non-high-risk APL patients, with a sustained remission and a higher safety compared with the combination of ATRA with chemotherapy 7, 8. Nowadays, ATO has become a new standard treatment of APL.

The efficacy of ATO in APL treatment is based on a dose-dependent dual effect. Many *in vitro* and *in vivo* studies showed that ATO induced partial differentiation at low concentration (0.1-0.5μM) and induced apoptosis at high concentration (1.0-2.0μM) in PML-RARα positive APL cells 9. The underlying mechanisms of ATO in APL treatment have been reported by many different groups. Some studies showed that ATO plays an important role in apoptosis through the mitochondrial pathway 10. Other studies showed that ATO directly binds to the PML moiety of PML-RARα fusion protein and induces degradation of PML-RARα via proteasomal pathway and autophagy 11. The degradation of PML-RARα triggers clearance of the promoters of target genes and promotes differentiation of APL cells. Meanwhile, the degradation of PML-RARα induces re-formation of PML nuclear bodies (NBs) and subsequently drives the activation of p53, senescence and APL clearance 1, 12.

Up to now, besides PML-RARα fusion gene, there are at least 13 RARα fusion genes involved in the pathogenesis of APL, which includes PLZF-RARα, NPM-RARα, NuMA-RARα, STAT5b-RARα, PRKAR1A-RARα, FIP1L1-RARα, BCoR-RARα, OBFC2A-RARα, TBLR1-RARα, GTF2I-RARα, IRF2BP2-RARα, FNDC3B-RARα, STAT3-RARα 13-16. APL harboring different RARα fusion genes exhibits different sensitivity to ATO treatment in case reports. Among them, APLs with PRKAR1A-RARα, GTF2I-RARα, IRF2BP2-RARα, FNDC3B-RARα and TBLR1-RARαare sensitive to ATO, but the rest of them exhibit poor response to ATO 2, 6. However, its underlying mechanism is poorly investigated. Though APLs with variants other than PML-RARα account for a small portion of the APL population, their prognosis is worse than APL with PML-RARα and appropriate management for these patients is still limited. Thus, further study of the treatment and underlying mechanism of the rare APLs is in need.

In our previous study, we first reported the TBLR1-RARα fusion gene (GenBankKF589333) in APL cases as the tenth RARα chimeric gene of APL 2. We found that ATRA could degrade TBLR1-RARα fusion protein and abrogate its homodimerization *in vitro*. ATRA also induced TBLR1-RARα dissociation and degradation of transcriptional corepressors, consequently transactivated RARα target genes and induced differentiation of TBLR1-RARα positive APL cells. However, according to our case, the APL patient with TBLR1-RARα had to suspend ATRA treatment on day 24 because of severe pulmonary infection. On day 32, bone marrow smear showed hypercellularity with 58% promyelocytes, which indicated the patient did not achieve CR. Eleven days after suspension, another course of treatment was conducted with ATO (10mg/d for 28 days) and Mitoxantrone (5mg/m^2^/d from days 6 to 8). Forty-six days after discontinuation of ATO, CR was documented 2. In this case, the APL patient with TBLR1-RARα was sensitive to ATO treatment. However, the biological response and its underlying mechanism of ATO treatment in TBLR1-RARα positive APL cells are still unclear.

In the present study, we identified that ATO induced proteasome-mediated degradation of TBLR1-RARα, leading to cell differentiation in TBLR1-RARα positive leukemia cells. We further demonstrated that ATO inhibited cell growth and promoted apoptosis in TBLR1-RARα positive cells through mitochondrial pathway. Our study attempts to provide clues for the management of TBLR1-RARα positive APL patients.

## Materials and methods

### Cell lines and reagents

HL-60 (acute promyelocytic leukemia cell line), U937 (acute myelomonocytic leukemia cell line), HEK293, CV-1 and 293T cells were purchased from the American Type Culture Collection (ATCC). HL-60 and U937 cells were routinely cultured in RPMI-1640 medium (Gibco) supplemented with 10% fetal bovine serum (HyClone). HEK293, CV-1 and 293T cells were maintained in Dulbecco's modified Eagle medium (Gibco) supplemented with 10% fetal bovine serum (HyClone). All cells were cultured at 37 °C in a humidified 5% CO_2_ incubator.

ATO (A1010) and MG132 (474790) were purchased from Sigma-Aldrich. CD11b (555388) and CD14 (562335) antibodies and IgG antibody (555749) were purchased from BD Pharmingen. The antibodies of anti-β-actin (4970), anti-p53 (2527), anti-Myc-tag (2276), anti-caspase-3 and anti-cleaved caspase-3 (9664), anti-caspase-9 and anti-cleaved caspase-9 (9502), anti-PARP and anti-cleaved-PARP (9542), anti-cytochrome (4272) and anti-flag (14792) were obtained from Cell Signaling Technology (CST, Beverly, Massachusetts, USA).

### Overexpression of TBLR1-RARα in HL-60 and U937

The lentivirus with pCDH1-MCS1-EF1-copGFP-TBLR1-RARα-Myc or pCDH1-MCS1-EF1-copGFP as vector control were kind gifts from Dr. Jianxiang Wang and Dr. Min Wang and were prepared as previously reported 2. The HL-60 and U937 cells were infected with prepared lentivirus. After infected for 72 hours, cells were sorted for GFP-positive cell populations by BD FACS Aria II System. The TBLR1-RARα expression was detected by real-time quantitative RT-PCR for mRNA level and Western blot for protein level using anti-Myc-tag antibody (CST 2276). Cells infected with pCDH1-MCS1-EF1-copGFP-TBLR1-RARα-Myc were named HL-60/TBLR1-RARα and U937/TBLR1-RARα. Cells infected with pCDH1-MCS1-EF1-copGFP were named HL-60/Vector and U937/Vector as vector controls.

### RNA isolation and real-time quantitative RT-PCR

HL-60 and U937 cells transfected with TBLR1-RARα or control vector were collected. RNA was isolated with Trizol reagent (Invitrogen, Carlsbad, CA, USA). cDNA was synthesized using a reverse transcription kit (Invitrogen). The real-time quantitative RT-PCR was conducted by a LongAmp® Taq PCR Kit (New England Biolabs). To amplify TBLR1-RARα, the following primers were used: 5'-ATGCCGTAATGCCTG ATG-3' and 5'-GAACTGCTGCTCTGGGTCT-3'. The PCR reaction was conducted under the following conditions: 5 minutes at 94 °C for denaturation, followed by 30 seconds at 94 °C, 30 seconds at 58 °C and 150 seconds at 72 °C for 28 cycles and 10 min at 72 °C for final elongation.

### Immunoblotting analysis

Protein lysate preparation was performed according to the manufacturer's instructions. The protein lysates were separated in 12% sodium dodecyl sulfate-polyacrylamide gel electrophoresis gels and transferred to Immobilon P Transfer Membrane (Millipore, Bedford, MA, USA). After regular blocking and washing, the membranes were sequentially incubated with primary antibodies overnight at 4 °C, and HRP-conjugated secondary antibodies for 1h at room temperature. The immunoreactive proteins were visualized using enhanced chemiluminescence detection reagents (Millipore, Billerica, MA, USA) and Image Quant LAS-4000 (Fujifilm, Tokyo, Japan). The protein quantification of the Western blot results was normalized to β-actin and then compared to the control group, which was normalized as 1.

### Colony formation assay

Cells were resuspended in IMDM medium (Gibco) (400 cells/mL) with 0.9% methyl cellulose, 30% FBS, 0.02 mM glutamine, 0.1 mM mercaptoethanol, 100 U/mL Streptomycin and treated with 4 μM ATO or without ATO as control. Then, cells were mixed by a syringe and seeded in a 12-well plate (1 mL cells for each well and three replicate wells for each group). After incubation at 37 °C in a 5% CO_2_ for 6 days, the colony was observed under an inverted microscope and the number of colonies with more than 50 cells was recorded. Colony formation rate (%) = number of colonies/number of incubated cells × 100%.

### Flow cytometry analysis

CD11b and CD14, markers for cell differentiation, were detected by flow cytometry. Cells were seeded at a density of 1×10^6^ cells per well in 6-well culture plates and treated with different concentrations (0 μM, 0.25 μM, 0.5 μM, 1 μM, 2 μM) of ATO. On days 0, 3, 6, 9 and 12 of treatment, the cells were collected and incubated with CD11b or CD14 for 30 minutes, and examined by BD FACS LSR II flow cytometer. The IgG antibody treated group was used as a negative control. The percentage of CD11b-positive cells or CD14-positive cells was analyzed using FlowJo software, version 7.6.1.

Apoptosis was detected by flow cytometry using Annexin V-PE/7-AAD Apoptosis Detection Kit (MultiSciences Biotech Co., Ltd, Hangzhou, China). Cells were seeded 4×10^5^ cells per well in 6-well culture plates and incubated with different concentrations (0 μM, 2 μM, 4 μM, 8 μM) of ATO for 24h or 48h. Then, cells were collected and incubated with AnnexinV-PE and 7-AAD according to the manufacturer's instructions. The cells were examined and analyzed as described above.

### Wright staining assay

Cells were seeded at a density of 2×10^5^ cells per well in 6-well plates and treated with 2 μM ATO or without ATO as control. After incubation at 37 °C in a 5% CO_2_ for 12 days, cells were collected, flaked on slides, and stained with Wright staining solution. Finally, the slides were observed under a microscope and cells in five random view fields were photographed.

### Cell viability

The cells were seeded at a density of 5×10^3^ cells per well in 96-well flat-bottom plates. After incubated overnight, the cells were treated with different concentrations of ATO for 72 h. Then, the cell viability was assessed through the colorimetric MTS assay. Briefly, 20 μl MTS solution (Promega) was added to each well, and the plates were continued to be incubated for 4 h at 37 °C. The absorbance value of each well was read at 490 nm using an ELISA plate reader instrument (BIO RAD, Model 680, Japan). The absorbance values with different concentrations of ATO were compared to that of the control group without ATO, which was assumed to be 100%. All of the experiments were performed using three wells per experiment and repeated at least three times.

### Luciferase assay

Luciferase assay was conducted as previous report 2. RARE Cignal reporter (Cignal Pathway ReporterKits; Qiagen) and respective vectors (RARα and TBLR1-RARα expression vectors, which were kind gifts from Dr. Jianxiang Wang, Dr. Min Wang and Dr. Takeshi Kondo, and were constructed as previously described 2) were transiently co-transfected into CV-1 cells or HEK293 cells. The cells were then incubated with different concentrations of ATO for 48 hours. Luciferase assay was conducted as previously reported 2. The ratio between firefly and Renilla luciferase was used to normalize the transfection efficiency.

### Intracellular ROS and mitochondria-specific superoxide measurement by flow cytometry

The intracellular ROS and mitochondria-specific superoxide levels were measured by flow cytometry. Briefly, 2×10^5^ cells were seeded in 60-mm dishes. After incubated for overnight, the cells were treated with 4 or 8 μM ATO for 24h or 48h. Then, cells were treated with 10 μM fluorescent probe 20',70'-dichlorodihydrofluo-rescein diacetate (DCFH-DA, Sigma, St. Louis, MO, USA) or 5 μM MitoSOX^TM^ Red mitochondrial superoxide indicator (M36008, Invitrogen) in dimethyl sulfoxide for 30 min in fresh medium in dark. After washing with PBS, the cells were collected by centrifugation, suspended in PBS and detected with the FL1 or FL2 channel of flow cytometry (FACSCalibur flow cytometer, BD, CA, USA) to measure intracellular ROS or mitochondrial superoxide. The values of cells with TBLR1-RARα were compared to those cells with vector, which were normalized to be 1. All of the experiments were repeated at least three times.

### Statistical analysis

At least three repetitions were performed for each experiment. Data were shown as the means ± SD. The significance of differences between two groups of normally distributed data was determined using Student's *t*-test. All statistical analyses were carried out using the SPSS 22.0 software package. P<0.05 was considered as statistically significant.

## Results

### Overexpression of TBLR1-RARα in HL-60 and U937 cell lines

**Figure 1:**
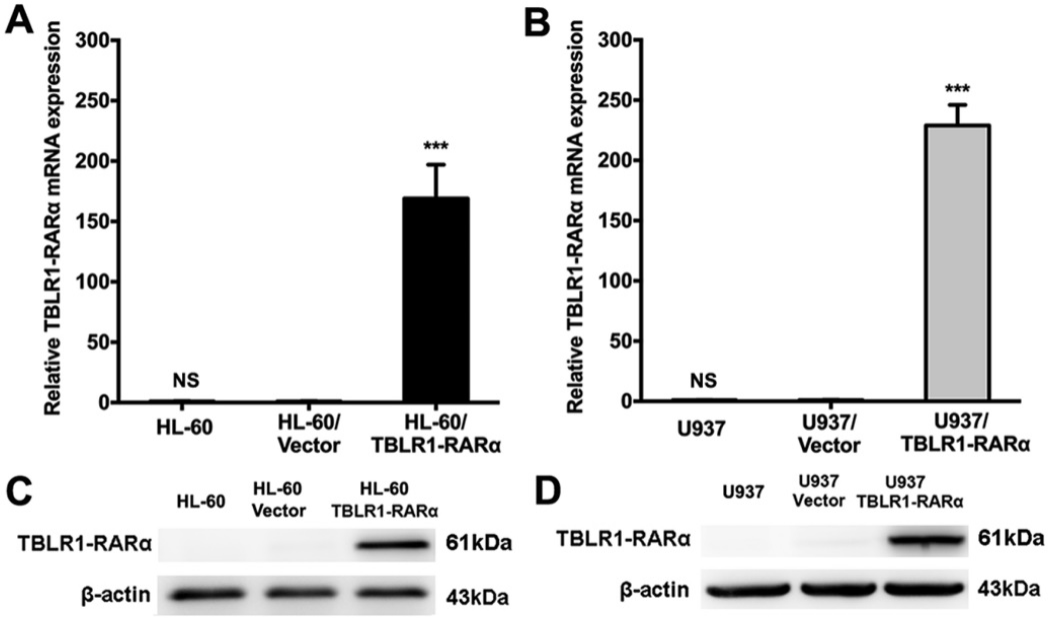
** The overexpression of TBLR1-RARα in HL-60 and U937 cells. (A and B)** The mRNA expression of TBLR1-RARα was detected by real-time quantitative PCR. GAPDH was used as internal control. The relative TBLR1-RARα mRNA level was determined using the the 2^(-ΔΔCt)^ method where ΔΔCt=ΔCt(TBLR1-RARα groups) - ΔCt(Vector groups) and ΔCt=(Ct TBLR1-RARα-CtGAPDH). Data are presented as the mean ± SEM (n=3) (***p <0.001). **(C and D)** The protein expression of TBLR1-RARα was detected by Western-blot. β-actin was used as internal control. The result is representative of three independent experiments.

HL-60 and U937 cells infected with either lentivirus pCDH1-MCS1-EF1-copGFP-TBLR1-RARα-Myc or pCDH1-MCS1-EF1-copGFP were constructed, respectively. Expression of TBLR1-RARα in these cell lines was detected by RT-qPCR and Western blot (Fig. 1). TBLR1-RARα mRNA and protein levels were both markedly increased in HL-60/TBLR1-RARα and U937/TBLR1-RARα compared with vector control. HL-60/Vector and U937/Vector cells showed almost no change in TBLR1-RARα expression compared with wild-type cells.

### TBLR1-RARα promotes ATO-induced cell proliferation inhibition in leukemia cells by enhancing apoptosis

**Figure 2:**
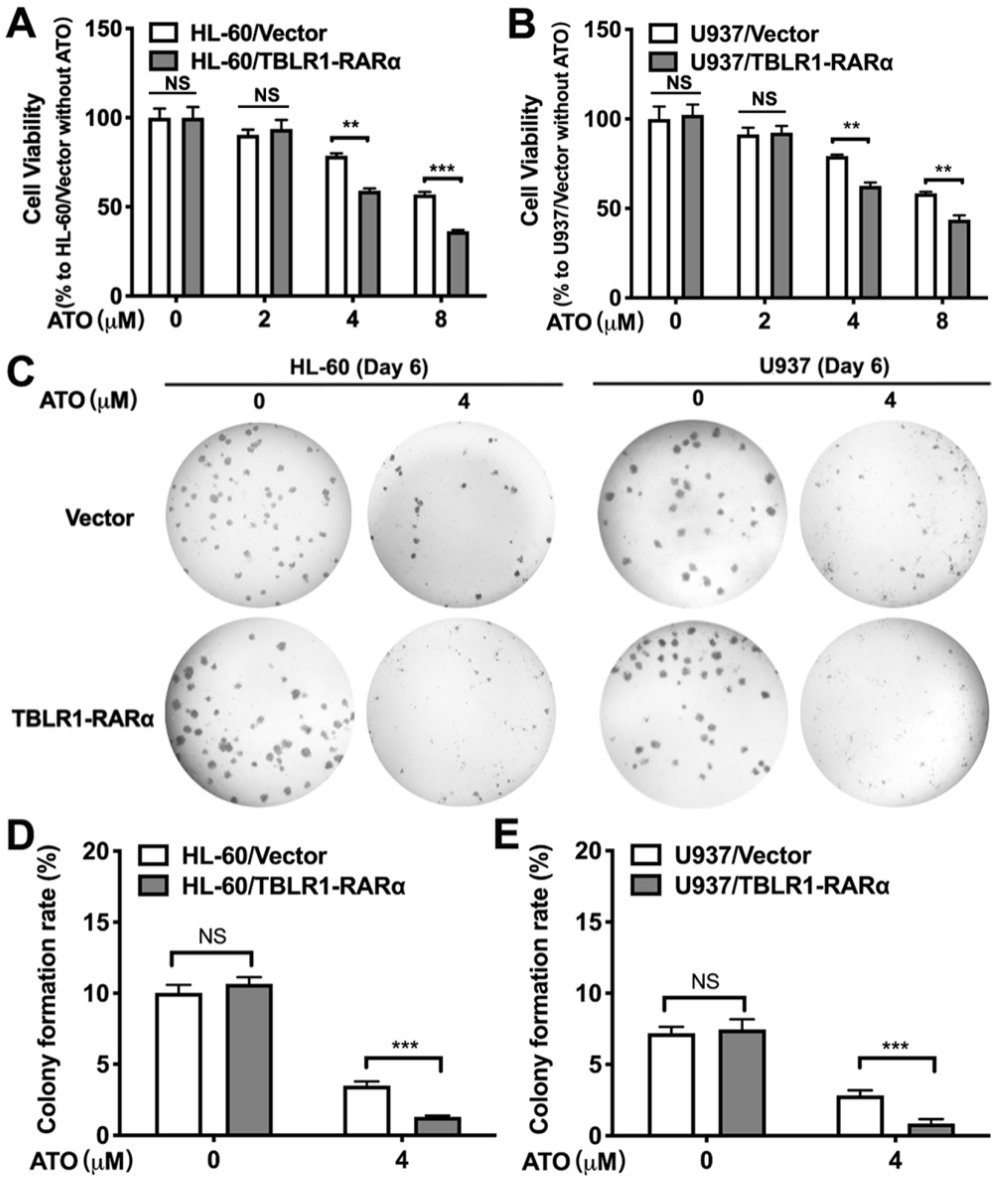
** TBLR1-RARα expression enhances ATO-induced inhibition of cell viability and colony forming efficiency in HL-60 and U937 cells. (A and B)** The cells were exposed to various concentrations of ATO for 72h, and cell viability was assessed by MTS. **(C)** The presentive results of the colony formation assay after 6 days treatment with ATO. **(D and E)** The quantitative results of the colony formation assay. Data are presented as the mean ± SEM (n=3) (NS=no significance, **p <0.01, ***p <0.001). The group of cells with vector was served as control group for each cell model. The absorbance values of each group were compared to that of control group with ddH_2_O, which was normalized as 100%.

To explore the role of TBLR1-RARα expression in cell proliferation inhibition of leukemia cells induced by ATO, we performed cell viability assay and colony formation assay in HL-60 and U937 cell models. The inhibition of cell viability induced by ATO exhibited both dose-dependent manner and time-dependent manner in HL-60 and U937 wild type cell lines (Supplementary Figure 1). However, HL-60/TBLR1-RARα and U937/TBLR1-RARα showed significantly higher inhibition of cell proliferation compared with control groups after ATO treatment for 72h, respectively (Fig.2A-B). The inhibition of cell proliferation induced by ATO exhibited a dose-dependent manner. In accordance with the results of cell viability, colony formation results showed that TBLR1-RARα over-expressed cell lines had a significant increase of colony formation inhibition after ATO treatment compared with the control groups (Fig. 2C-E).

**Figure 3:**
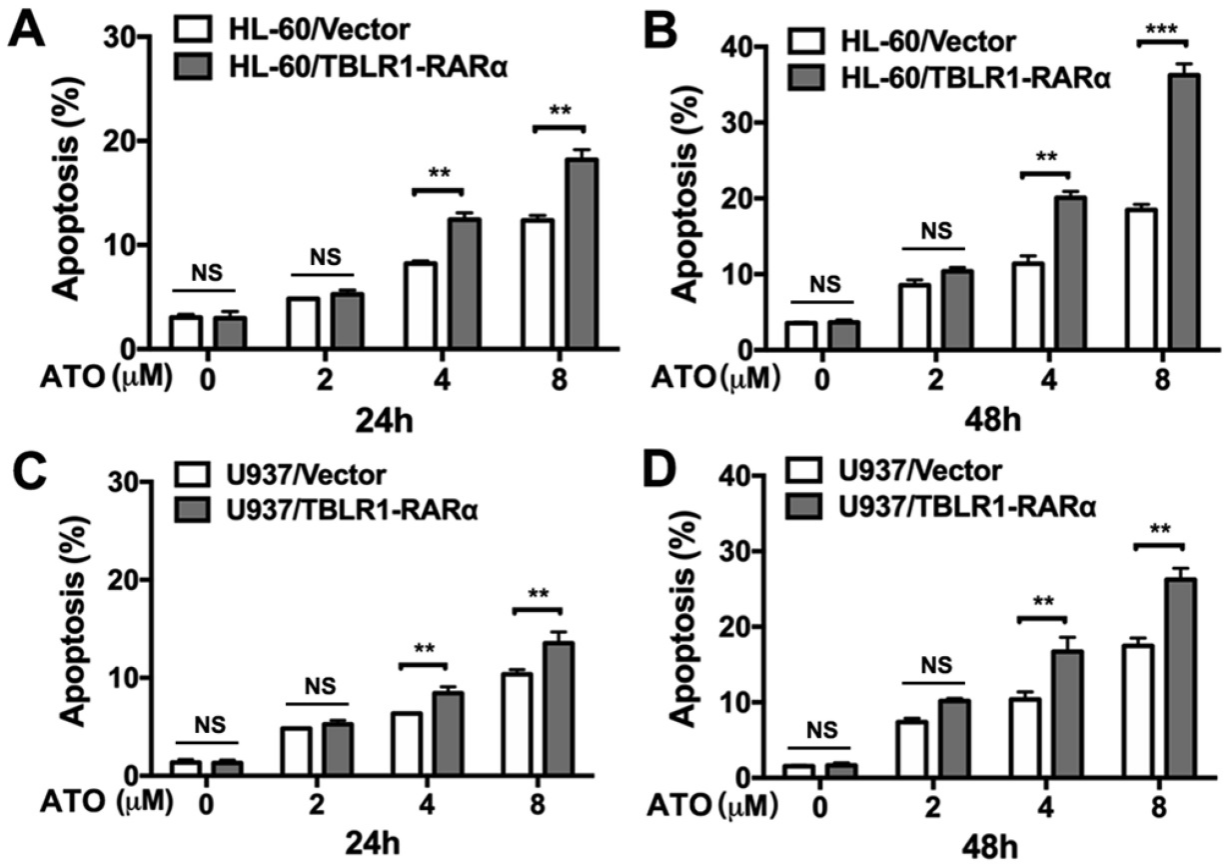
** TBLR1-RARα expression promotes apoptosis induced by ATO in HL-60 and U937 cells. (A and B)** The apoptosis in HL-60 cell model was detected by flow cytometry with various concentrations of ATO treatment for 24h or 48h, respectively. **(C and D)** The apoptosis in U937 cell model was detected by flow cytometry with various concentrations of ATO treatment for 24h or 48h, respectively. Data are presented as the mean ± SEM (n=3) (**p <0.01, ***p <0.001). The group of cells with vector was served as control group for each cell model.

In order to further determine the role of TBLR1-RARα expression in the enhancement of ATO-induced cell apoptosis, apoptosis assay was conducted in both cell models by flow cytometry. As shown in Fig. 3A-B, HL-60/TBLR1-RARα cells showed higher apoptosis proportion than HL-60/ Vector cells at both 24 and 48 hours after ATO treatment. Consistent with the result in HL-60 cell model, U937/TBLR1-RARα cells also exhibited a higher proportion of apoptosis at both 24 and 48 hours after ATO treatment (Fig. 3C-D). These results indicated that TBLR1-RARα enhances ATO-induced apoptosis and promotes cell proliferation inhibition in leukemia cells *in vitro*.

### TBLR1-RARα promotes ATO-induced cell differentiation in leukemia cells

**Figure 4:**
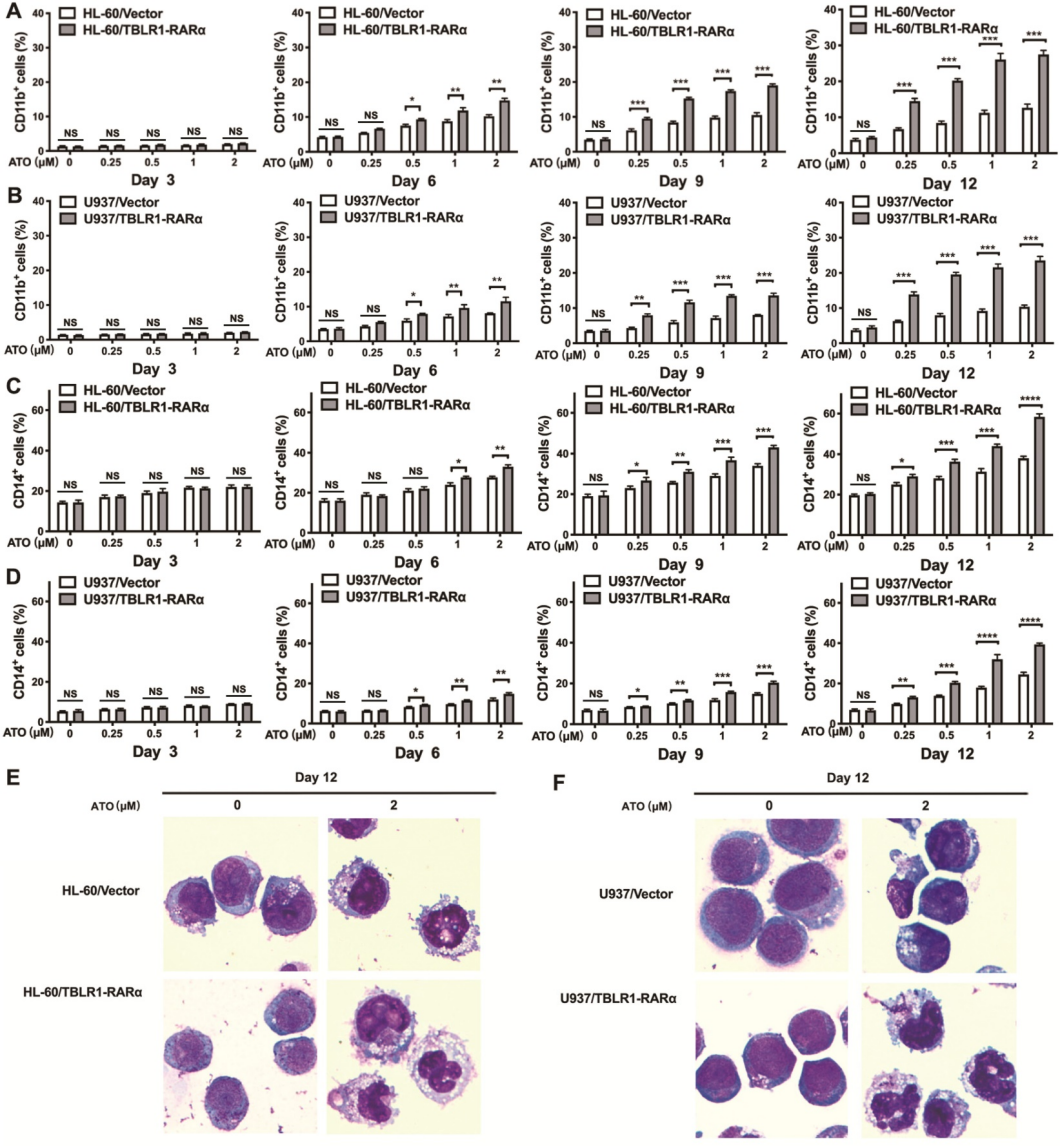
** TBLR1-RARα expression increases ATO-induced cell differentiation in HL-60 and U937 cells. (A, B)** The cell surface marker CD11b expression levels in HL-60 and U937 cell models were detected by flow cytometry with various concentrations of ATO treatment for 3, 6, 9 and 12 days. **(C, D)** The cell surface marker CD14 expression levels in HL-60 and U937 cell models were detected by flow cytometry with various concentrations of ATO treatment for 3, 6, 9 and 12 days. **(E)** The presentive results of Wright staining for HL-60 cell models with ATO treatment for 12 days. (**F**) The presentive results of Wright staining for U937 cell models with ATO treatment for 12 days. Data are presented as the mean ± SEM (n=3) (*p <0.05, **p <0.01, ***p <0.001). The group of cells with vector was served as control group for each cell model.

To examine whether TBLR1-RARα could induce ATO-mediated cell differentiation in leukemia cells, flow cytometry and Wright staining assay were conducted. HL-60/TBLR1-RARα, HL-60/Vector and U937/TBLR1-RARα, U937/Vector cells were treated with ATO and cell surface marker CD11b and CD14 expression levels were detected by flow cytometry. As shown in Fig. 4A, C, the proportions of CD11b-positive and CD14-positive cells were both significantly higher in HL-60/TBLR1-RARα cells than those in HL-60/ Vector cells at different time points after ATO treatment. As the same trend, U937/TBLR1-RARα cells also had higher proportions of CD11b-positive and CD14-positive cells compared to the control group at different time points after ATO treatment (Fig. 4B, D). The expression of CD11b and CD14 induced by ATO in TBLR1-RARα positive cells was found in a time and dose-dependent manner. In accordance with the results of the flow cytometry assay, cell morphology observation showed that HL-60/TBLR1-RARα cells and U937/TBLR1-RARα cells exhibited certain differentiated characteristics after ATO treatment compared with the control group, respectively (Fig. 4E, F). Together, these results indicated that TBLR1-RARα promotes ATO-induced cell differentiation in leukemia cells *in vitro*.

### TBLR1-RARα promotes ATO-induced cell apoptosis via mitochondrial pathway in leukemia cells

**Figure 5:**
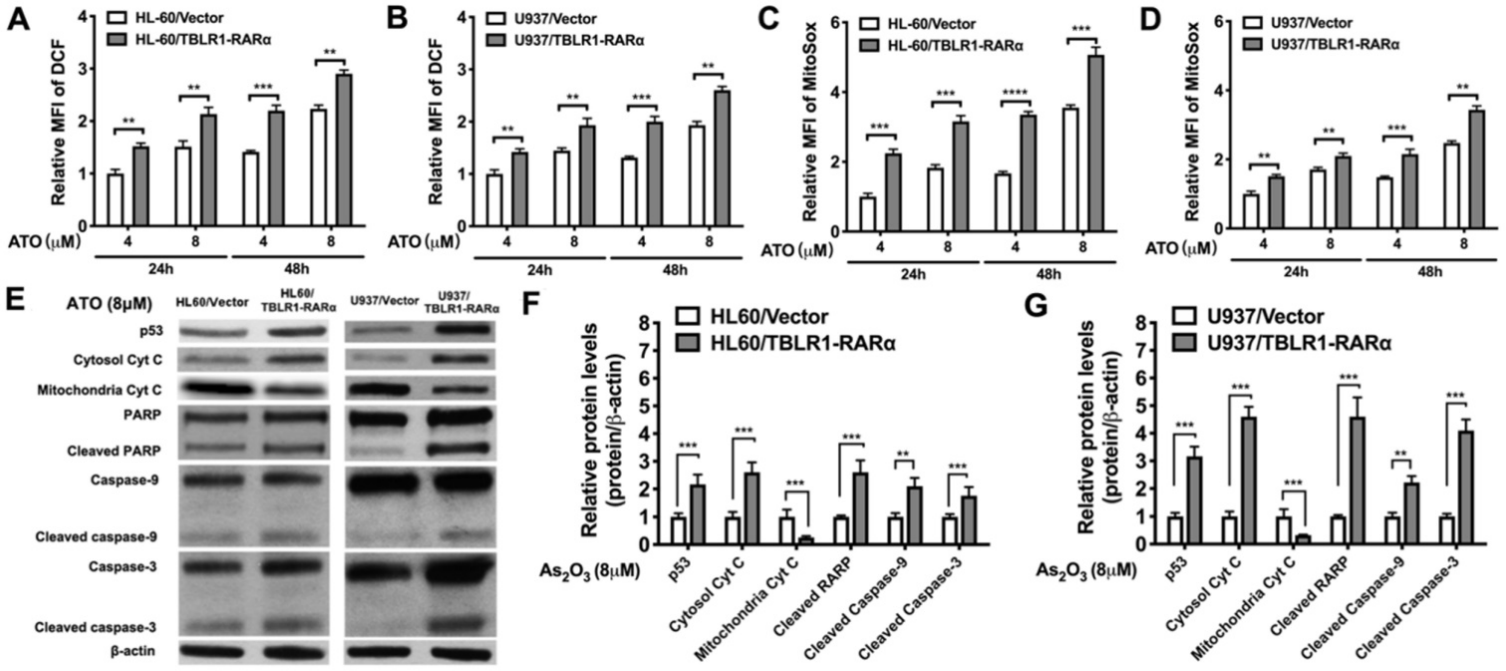
** TBLR1-RARα promotes ATO-induced cell apoptosis via mitochondrial pathway in leukemia cells. (A and B)** The DCF levels indicating intracellular ROS in HL-60 and U937 cell models were detected by flow cytometry at 24 and 48 h of ATO treatment. **(C and D)** The MitoSox levels indicating mitochondrial superoxide in HL-60 and U937 cell models were detected by flow cytometry at 24 and 48 h of ATO treatment.** (E)** The mitochondrial pathway related proteins were detected by Western blot in both cell models. β-actin was used as internal control.** (F and G)** The quantitative results of Western blot for HL-60 and U937 cell models, respectively. β-actin was used as internal control. Data are presented as the mean ± SEM (n=3) (**p <0.01, ***p <0.001). The group of cells with vector was served as control group for each cell model. The protein levels of each group were compared to that of control group, which was normalized as 1.

To further explore the mechanism of TBLR1-RARα on enhancing ATO-induced apoptosis and inhibiting cell proliferation, we examined ROS levels by DCF and MitoSox assays and apoptosis-related proteins by Western blot in both cell models. The results showed that ATO treatment increased the ROS levels in both control groups. The same trends were found both in the results of DCF and MitoSox assays. However, the ROS levels in TBLR1-RARα positive cells were significantly higher than those in control groups at both 24 h and 48 h after ATO treatment (Fig. 5A-D). Furthermore, apoptosis-related proteins p53, cleaved PARP, cleaved caspase-9 and cleaved caspase-3 levels in TBLR1-RARα positive cells were increased compared with those in the control groups, which also indicated that TBLR1-RARα promoted ATO-induced apoptosis through mitochondria pathway (Fig. 5E-G). Moreover, we examined cytochrome c levels in cytosol and mitochondria. The cytochrome c levels in mitochondria of TBLR1-RARα positive cells were lower than those in control groups, while the cytochrome c levels in cytosol of TBLR1-RARα positive cells were higher than those in control groups (Fig. 5E-G). These results suggested that TBLR1-RARα enhanced the ATO-induced release of cytochrome c from mitochondria to cytosol. Together, these results indicated that TBLR1-RARα enhances ATO-induced apoptosis in leukemia cells *in vitro* through the mitochondria pathway.

### ATO induces TBLR1-RARα degradation via proteasome pathway to promote cell differentiation in leukemia cells

**Figure 6:**
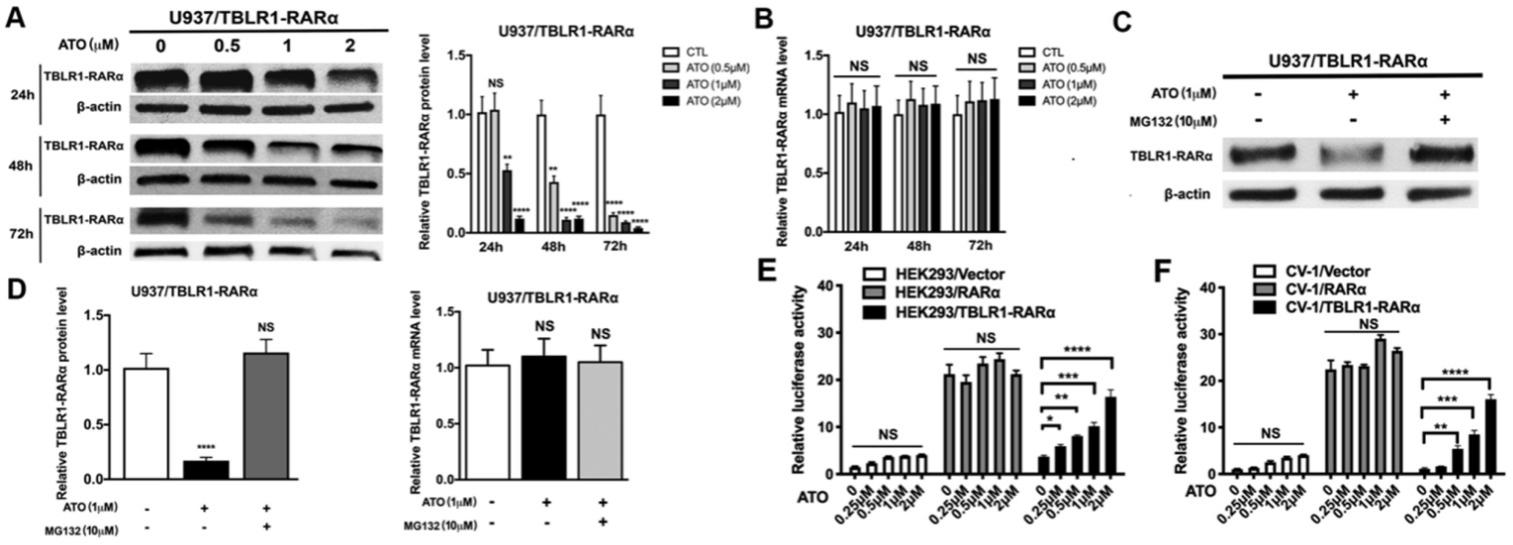
** ATO induces proteasome dependent TBLR1-RARα degradation to promote transcriptional reactivation. (A and B)** The TBLR1-RARα protein and mRNA levels in U937/ TBLR1-RARα cells with various concentrations of ATO treatment were detected by Western blot and real-time PCR, respectively. **(C and D)** The TBLR1-RARα protein and mRNA levels in U937/ TBLR1-RARα cells with ATO and MG132 were detected by Western blot and real-time PCR, respectively. The group of cells without both ATO and MG132 treatment were served as the control group. The protein and mRNA levels of each group were compared to that of control group, which was normalized as 1. **(E and F)** The transcriptional reactivation level was detected in HEK293 and CV1 cells by luciferase assay, respectively. The group of cells without ATO was served as control group. The group of cells with vector were served as control group for each cell model. Data are presented as the mean ± SEM (n=3) (*p <0.05, **p <0.01, ***p <0.001). The relative luciferase activity levels were compared to that of control group without ATO treatment, which was normalized as 1.

The degradation of PML-RARα fusion protein plays a critical role in the long-term remission of PML-RARα positive APL 6. To examine the role of ATO in TBLR1-RARα degradation in leukemia cells, the expression of TBLR1-RARα was detected after ATO treatment. TBLR1-RARα protein level was decreased at the presence of ATO in a time and dose-dependent manner (Fig. 6A). However, there is no significant difference in mRNA levels of TBLR1-RARα among each group with or without ATO treatment for 48 h (Fig. 6B). To further assess whether proteasome pathway was involved in the degradation of TBLR1-RARα, proteasome-dependent degradation pathway inhibitor MG132 (10 µM) was used to treat TBLR1-RARα positive cell models with or without ATO (1 µM) treatment. As shown in Fig. 6C and D, the protein level of TBLR1-RARα was decreased at the presence of ATO treatment, while there is no significant decrease at presence of both MG132 and ATO treatment. Meanwhile, the mRNA levels of TBLR1-RARα were similar among each group (Fig. 6D). These results indicated that ATO increased TBLR1-RARα protein degradation via the proteasome pathway. The degradation of PML-RARα fusion protein is vital for the clearance of promoters of target genes, which are involved in activation of cell differentiation induced by ATO in PML-RARα positive APL 1, 17. Transcriptional activation of RARα target genes through RAREs is the key action in ATO and ATRA induced cell differentiation 17, 18. To elucidate the role that TBLR1-RARα plays in enhancing ATO-induced cell differentiation, luciferase assays were conducted. In response to ATO treatment, TBLR1-RARα exhibited partially transcriptional reactivation induced by ATO in a dose dependent manner compared with RARα group (Fig. 6E-F). Altogether, ATO induces TBLR1-RARα degradation by the proteasome pathway to promote cell differentiation in leukemia cells.

## Discussion

Although PML-RARα positive APL counts for the vast majority of APL, at least 13 other types of RARα fusion genes have been reported involved in the pathogenesis of APL 6. APLs harboring different RARα fusion genes exhibit a different response to standard APL treatment and most of them have a worse prognosis except PML-RARα positive APL. Hence, further studies to explore proper drug treatment on APL without classic PML-RARα fusion gene are in great need, which will help to improve the survival rate and prognosis of APL. TBLR1-RARα fusion gene was the tenth RARα chimeric gene of APL reported by our groups in our previous work 2. We previously reported 3 cases of APL harboring TBLR1-RARα fusion gene. Several groups have reported cases of APL involving t(3;17) chromosomal translocation 19, 20. Most recently, a study explored the molecular features of APL with variant RARα fusion genes and showed that TBLR1-RARα fusion gene was detected in one of 19 cases with alternative RARα or RARG fusion genes 21. These studies suggested that APL cases with TBLR1-RARα fusion gene were recurrent. According to the clinical features of the cases with TBLR1-RARα fusion gene, APL patients with TBLR1-RARα fusion gene are resistant to the ATRA treatment and have a relatively poor prognosis 2, 6, 21. However, in our previous case report, we observed one APL patient with TBLR1-RARα fusion gene achieved CR after receiving ATO combined with chemotherapy, indicating that APL with TBLR1-RARα fusion gene may be sensitive to ATO treatment 2. To elucidate the possible treatment for TBLR1-RARα positive APL and identify several clues for future clinical strategy, the biological response of TBLR1-RARα positive APL cells to ATO treatment and the mechanism involved was investigated in the present study.

Consistent with clinical observation, we found that TBLR1-RARα promotes ATO-induced differentiation, proliferation inhibition and apoptosis. Furthermore, our results showed that lower concentration (0.25-2.0 μM) of ATO induced differentiation of TBLR1-RARα positive APL cells after a long-time culture, whereas higher concentration (4.0-8.0 μM) of ATO induced apoptosis of TBLR1-RARα positive APL cells. Similar to the effect of ATO in TBLR1-RARα positive APL cells, studies showed that ATO induced partial differentiation and apoptosis of PML-RARα positive APL cells on a dose-dependent dual effect 10. *In vitro* studies showed that lower concentration (0.1-0.5 μM) of ATO induced partial differentiation after a long-time culture, while higher concentration (1.0-2.0 μM) of ATO induced apoptosis in PML-RARα positive APL cells 5. Although our results and the results found in PML-RARα positive APL showed that ATO has a similar dose-dependent dual effect on both cells, the ATO concentration required for differentiation and apoptosis of TBLR1-RARα positive APL cells was higher than that was needed for PML-RARα positive APL cells. These results indicate that TBLR1-RARα positive APL cells are sensitive to ATO treatment *in vitro*. However, they are less sensitive to ATO treatment compared with PML-RARα positive APL cells. Our results showed that TBLR1-RARα enhanced ATO-induced leukemia elimination *in vitro*, which may explain why the TBLR1-RARα positive APL patient achieved CR by using ATO plus chemotherapy. Nevertheless, compared with PML-RARα positive APL, TBLR1-RARα positive APL is less sensitive to ATO treatment. Therefore, the prognosis of TBLR1-RARα positive APL patients was relatively worse.

Degradation of RARα fusion protein plays a vital role in ATO mediated APL eradication and it is highly related to differentiation and apoptosis of APL cells. As an E3 ubiquitin ligase, TBLR1 involves in protein degradative polyubiquitination 22. Interestingly, TBLR1 is a component of N-CoR/SMRT-TBLR1 corepressor complex and functions as a transcriptional corepressor in the absence of ligands for nuclear receptors. Meanwhile, in the presence of ligands, TBLR1 recruits ubiquitin/proteasome machinery to degrade the co-repressors, which plays a key role in transcriptional activation 23. The N-terminal F-box domain of TBLR1, which is essential for the ubiquitin-mediated protein degradation via the proteasome pathway, also exists in TBLR1-RARα 2, 24. Therefore, we further investigated the role of proteasome pathway in ATO-induced TBLR1-RARα degradation. In the present study, TBLR1-RARα was degraded by ATO treatment via the proteasome pathway in a time and dose-dependent manner. Further luciferase assay indicated that degradation of TBLR1-RARα partially reactivated RARα target genes and consequently promoted differentiation of TBLR1-RARα positive APL cells. In accordance with our results, dual biological function induced by degradation of PML-RARα accounts for long-term remission of ATO treatment 4. ATO directly binds to the PML moiety of PML-RARα fusion protein and results in PML-RARα degradation through the proteasome pathway 1, 25. As a result, removal of PML-RARα fusion protein leads to clearance of the promoters of RARα target genes and further triggers differentiation of APL cells 17. Moreover, our results showed that ATO induces activation of p53, PARP, caspase-9 and caspase-3, and also promotes the release of ROS and cytochrome c in TBLR1-RARα positive leukemia cells. Similar to the therapeutic function in TBLR1-RARα positive APL, ATO induced mitochondrial transmembrane potential collapse, PTPC opening, cytochrome c and ROS release, and caspases activation in PML-RARα positive APL 26. Besides, the destruction of PML-RARα induces re-formation of PML nuclear bodies and activates p53, which further activates senescence and APL clearance 12, 27. The above results indicated that the mitochondrial pathway plays a critical role in ATO-induced apoptosis of TBLR1-RARα positive leukemia cells.

The current study gained some insight into the biological function and underlying mechanism of ATO treatment in TBLR1-RARα positive leukemia cells *in vitro*. However, further study is needed to elucidate the molecular mechanism involved in ATO-induced degradation of TBLR1-RARα. To our current knowledge, autophagic-lysosomal pathway may involve in ATO-induced degradation of TBLR1-RARα and further investigation is undergoing (data not show). Moreover, efficacy of ATO in TBLR1-RARα positive APL *in vivo* needs further investigation.

## Conclusions

In summary, TBLR1-RARα enhances ATO-induced differentiation, proliferation inhibition and apoptosis in leukemia cells in a time and dose dependent manner. Further mechanism investigation indicated that the mitochondrial pathway was involved in ATO-induced apoptosis of TBLR1-RARα positive leukemia cells. Moreover, TBLR1-RARα fusion protein was degraded via the proteosome pathway and resulted in reactivation of RARα target gene, which leads to ATO-induced differentiation. Current study provides evidence that ATO may be a promising drug for TBLR1- RARα positive APL treatment.

## Supplementary Material

Supplementary figure.

## Abbreviations

AML: acute myeloid leukemia
APL: Acute promyelocytic leukemia
ATCC: American Type Culture Collection
ATO: arsenic trioxide
ATRA: all-trans retinoic acid
CR: complete remission
CST: Cell Signaling Technology
DCF: dichlorofluorescein
DCFH-DA: dichlorodihydrofluo-rescein diacetate
PML: promyelocytic leukemia
RARα: retinoic acid receptor alpha
NBs: nuclear bodies
