# Supplementary material for: Arsenic trioxide induces proteasome dependent TBLR1-RARα degradation to improve leukemia eradication through cell differentiation enhancement
Source: J Cancer. 2022 Apr 18;13(7):2301–11. doi: 10.7150/jca.66175 (PMC9066217; doi:10.7150/jca.66175)
Supplement: Supplementary file 1 — Supplementary figure. [file jcav13p2301s1.pdf]

Supplementary Figure 1

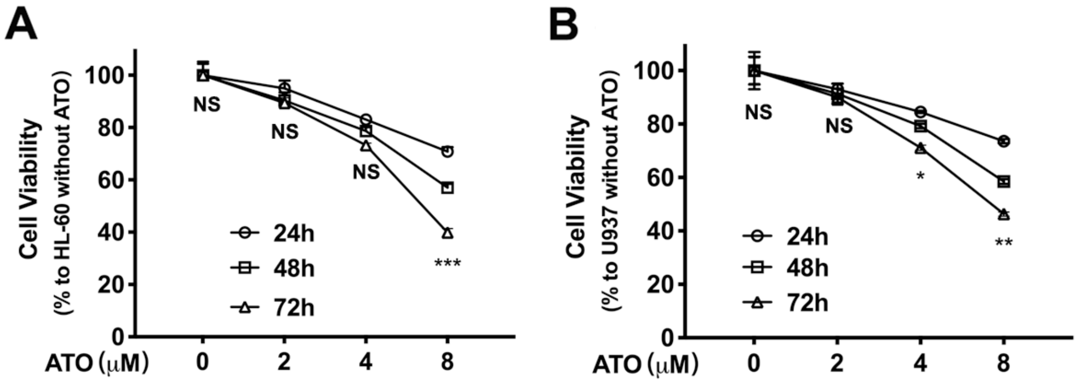

Figure S1. ATO induced the inhibition of cell viability in HL-60 and U937 wild type cells. (A) and **(B)** The cells were exposed to various concentrations of ATO for 24h, 48h and 72h, and cell viability was assessed by MTS. Data are presented as the mean ± SEM (n=3) (NS=no significance, *p <0.05, **p <0.01, ***p <0.001). The absorbance values of each group were compared to that of control group with ddH_2O, which was normalized as 100%.
